# Diagnosis and Screening Strategies for Detection of Familial Hypercholesterolaemia in Children and Adolescents in Italy: A Survey from the LIPIGEN Paediatric Group

**DOI:** 10.3390/children12030288

**Published:** 2025-02-26

**Authors:** Cristina Pederiva, Federica Galimberti, Manuela Casula, Giuseppe Banderali, Guglielmo Beccuti, Vanessa Bianconi, Giacomo Biasucci, Marta Biolo, Marco Bucci, Paola Sabrina Buonuomo, Paolo Calabrò, Stefano Carugo, Angelo Baldassare Cefalù, Nadia Citroni, Nicholas Cocomello, Sergio D’Addato, Simona Gatti, Simonetta Genovesi, Ornella Guardamagna, Gabriella Iannuzzo, Lorenzo Iughetti, Giuseppe Mandraffino, Lorenzo Maroni, Ilenia Minicocci, Giuliana Mombelli, Tiziana Montalcini, Sara Moriglia, Sandro Muntoni, Fabio Nascimbeni, Angelina Passaro, Fabio Pellegatta, Livia Pisciotta, Elena Sani, Francesco Sbrana, Roberto Scicali, Patrizia Suppressa, José Pablo Werba, Maria Grazia Zenti, Marcello Arca, Maurizio Averna, Sebastiano Calandra, Alberico Luigi Catapano, Patrizia Tarugi, Maria Elena Capra

**Affiliations:** 1 Clinical Service for Dyslipidaemias, Study and Prevention of Atherosclerosis in Childhood, Paediatrics Unit, ASST-Santi Paolo e Carlo, 20142 Milan, Italy; cristina.pederiva@asst-santipaolocarlo.it (C.P.); giuseppe.banderali@asst-santipaolocarlo.it (G.B.); 2 IRCCS MultiMedica, 20099 Sesto San Giovanni, Italy; federica.galimberti@multimedica.it (F.G.); alberico.catapano@unimi.it (A.L.C.); 3 Epidemiology and Preventive Pharmacology Service (SEFAP), Department of Pharmacological and Biomolecular Sciences, University of Milan, 20133 Milan, Italy; 4 Division of Endocrinology, Diabetology and Metabolism, Department of Medical Sciences, University of Turin, 10126 Turin, Italy; guglielmo.beccuti@unito.it; 5 Unit of Internal Medicine, Department of Medicine and Surgery, University of Perugia, 06132 Perugia, Italy; vanessa.bianconi@unipg.it; 6 Centre for Paediatric Dyslipidaemias, Paediatrics and Neonatology Unit, Guglielmo da Saliceto Hospital, 29121 Piacenza, Italy; g.biasucci@ausl.pc.it (G.B.); m.capra@ausl.pc.it (M.E.C.); 7 Department of Medicine and Surgery, University of Parma, 43121 Parma, Italy; 8 Department of Medicine DIMED, University of Padova, 35128 Padua, Italy; marta.biolo@aopd.veneto.it; 9 Institute of “Clinica Medica”, Department of Medicine and Aging Science, “G. D’Annunzio” University of Chieti, 66100 Chieti, Italy; marco.bucci@unich.it; 10 Rare Disease and Medical Genetics Unit, Bambino Gesù Children’s Hospital IRCCS, 00163 Rome, Italy; psabrina.buonuomo@opbg.net; 11 Division of Clinical Cardiology, A.O.R.N. Sant’Anna e San Sebastiano, 81100 Caserta, Italy; paolo.calabro@unicampania.it; 12 Department of Translational Medical Sciences, University of Campania “Luigi Vanvitelli”, 80131 Naples, Italy; 13 Department of Clinical Sciences and Community Health, University of Milan, 20122 Milan, Italy; stefano.carugo@policlinico.mi.it; 14 Cardiology Unit, Department of Internal Medicine, Fondazione IRCCS Ca’ Granda Ospedale Maggiore Policlinico of Milan, 20122 Milan, Italy; 15 Department of Health Promotion, Mother and Child Care, Internal Medicine and Medical Specialties, University of Palermo, 90127 Palermo, Italy; abaldassare.cefalu@unipa.it (A.B.C.); maurizio.averna@unipa.it (M.A.); 16 Centro Dislipidemie e Aterosclerosi, Ospedale di Trento, APSS-Trento, 38122 Trento, Italy; nadia.citroni@apss.tn.it; 17 Department of Clinical Internal, Anaesthesiologic and Cardiovascular Sciences, Sapienza University of Rome, 00185 Rome, Italy; nicholas.cocomello@uniroma1.it; 18 AO Policlinico Umberto I, 00161 Rome, Italy; marcello.arca@uniroma1.it; 19 UO di Medicina Interna Cardiovascolare, Ambulatorio Dislipidemie, Università di Bologna, 40138 Bologna, Italy; sergio.daddato@unibo.it; 20 IRCCS S Orsola, 40138 Bologna, Italy; 21 Department of Pediatrics, Polytechnic University of Marche, 60123 Ancona, Italy; simona.gatti@ospedaliriuniti.marche.it; 22 School of Medicine and Surgery, University of Milano-Bicocca, 20126 Milan, Italy; simonetta.genovesi@unimib.it; 23 Istituto Auxologico Italiano IRCCS, 20149 Milan, Italy; 24 Paediatric Endocrinology, Department of Public Health and Paediatric Sciences, Turin University, 10126 Turin, Italy; ornella.guardamagna@unito.it; 25 Department of Clinical Medicine and Surgery, University of Naples “Federico II”, 80131 Naples, Italy; gabriella.iannuzzo@unina.it; 26 Pediatric Unit, Department of Medical and Surgical Sciences of the Mother, Children and Adults, University of Modena and Reggio Emilia, 41124 Modena, Italy; iughetti.lorenzo@unimore.it; 27 Department of Clinical and Experimental Medicine, Internal Medicine Unit, Lipid Center, University Hospital G. Martino, University of Messina, 98100 Messina, Italy; giuseppe.mandraffino@unime.it; 28 Ambulatorio Ipertensione Dislipidemie, UO Medicina Generale, ASST Valle Olona, Ospedale di Gallarate, 21013 Gallarate, Italy; lorenzo.maroni@asst-valleolona.it; 29 Department of Translational and Precision Medicine, Sapienza University of Rome, 00185 Rome, Italy; ilenia.minicocci@uniroma1.it; 30 Centro Dislipidemie, ASST Grande Ospedale Metropolitano Niguarda, 20162 Milan, Italy; giuliana.mombelli@ospedaleniguarda.it; 31 Department of Experimental and Clinical Medicine, University Magna Grecia, 88100 Catanzaro, Italy; tmontalcini@unicz.it; 32 Internal and Geriatric Medicine, University Politecnica delle Marche, 60126 Ancona, Italy; s.moriglia@inrca.it; 33 IRCCS-INRCA, Ancona 60124, Italy; 34 Department of Biomedical Science, University of Cagliari, 09124 Cagliari, Italy; smuntoni@unica.it; 35 Centre for Metabolic Diseases and Atherosclerosis, The ME.DI.CO. Association, 09123 Cagliari, Italy; 36 Department of Maternal-Infantile and Adult Medical and Surgical Sciences, University of Modena and Reggio Emilia, 41124 Modena, Italy; fabio.nascimbeni83@gmail.com; 37 Internal Metabolic Medicine Unit, Baggiovara Hospital, AOU of Modena, 41126 Modena, Italy; 38 Department of Translational Medicine, University of Ferrara, 44121 Ferrara, Italy; angelina.passaro@unife.it; 39 Center for the Study and Treatment of Metabolic Diseases, Atherosclerosis, and Clinical Nutrition, University Hospital of Ferrara Arcispedale Sant’Anna, 44124 Ferrara, Italy; 40 Centro per lo Studio dell’Aterosclerosi, Ospedale E Bassini, 20092 Cinisello Balsamo, Italy; fabio.nascimbeni@unimore.it; 41 Department of internal Medicine, University of Genoa, 16132 Genoa, Italy; livia.pisciotta@unige.it; 42 IRCCS Polyclinic Hospital San Martino, Operative Unit of Dietetics and Clinical Nutrition, 16132 Genoa, Italy; 43 Division of Endocrinology, Diabetes and Metabolism, Department of Medicine, University Hospital of Verona, 37134 Verona, Italy; elena.sani@aovr.veneto.it; 44 Lipoapheresis Unit, Reference Center for Diagnosis and Treatment of Inherited Dyslipidemias, Fondazione Toscana “Gabriele Monasterio”, 56124 Pisa, Italy; ifcfsbrana@ftgm.it; 45 Department of Clinical and Experimental Medicine, Internal Medicine, Garibaldi-Nesima Hospital, University of Catania, 95122 Catania, Italy; roberto.scicali@unict.it; 46 Department of Medicine and Surgery, LUM University Giuseppe Degennaro, 70010 Casamassima, Italy; suppressa@lum.it; 47 Atherosclerosis Prevention Unit, Centro Cardiologico Monzino IRCCS, 20138 Milan, Italy; pablo.werba@cardiologicomonzino.it; 48 Diabetes and Metabolism Unit, Pederzoli Hospital, Casa di Cura Privata, 37019 Peschiera del Garda, Italy; mariagrazia.zenti@ospedalepederzoli.it; 49 Institute of Biophysics (IBF), National Research Council (CNR), 90146 Palermo, Italy; 50 Department of Biomedical, Metabolic and Neural Sciences, University of Modena and Reggio Emilia, 41125 Modena, Italy; sebcal@unimore.it; 51 Department of Life Sciences, University of Modena and Reggio Emilia, 41125 Modena, Italy; patriziamaria.tarugi@unimore.it

**Keywords:** familial hypercholesterolemia, paediatric, screening, diagnosis, survey

## Abstract

Background: Awareness, diagnosis, and treatment of familial hypercholesterolemia (FH) starting from childhood are a cornerstone of cardiovascular disease prevention. The LIPIGEN Paediatric Group, a network of specialised centres for the diagnosis and management of familial genetic dyslipidemia, is an active part of this mission. Materials and Methods: This is the second exploratory survey organised within the LIPIGEN (LIpid transPort disorders Italian GEnetic Network) paediatric centres. A digital questionnaire consisting of 16 questions was proposed to the principal investigators of 35 LIPIGEN centres in September 2023. We analysed the main FH screening strategies implemented in Italy, which are the referral characteristics to the lipid clinics and clinical and biochemical criteria considered to diagnose FH in paediatric patients. Results: Centres frequently reported conducting cascade screening (88.6%) and reverse screening (57.1%), whereas 28.6% of respondents indicated using selective screening and only 5.7% reported employing child–parent screening. We documented a detailed biochemical characterisation of paediatric patients (62.9% of respondents usually perform full lipoprotein profile and 80% determine lipoprotein(a) for each patient) and a high percentage of genetic analysis (82.9%). We have also highlighted a quite low awareness of FH as a genetic condition involving paediatric patients among primary care paediatricians and general practitioners. Conclusions: The results of our survey show that specialised lipid centres usually have good diagnostic competence when dealing with paediatric patients with hypercholesterolemia. However, FH awareness and the importance of early diagnosis and treatment initiation in childhood still need to be further improved.

## 1. Introduction

Heterozygous familial hypercholesterolemia (FH) is a common genetic disease with an autosomal co-dominant inheritance. Its estimated prevalence worldwide is 1:250–300 in the general population, even if it is underestimated and undertreated in many countries [1,2,3]. Subjects with FH have elevated plasma total cholesterol and low-density-lipoprotein cholesterol (LDL-C) since the very first years of life, so if they are undetected and untreated, they develop premature atherosclerosis [4,5,6,7]. Early detection and treatment of subjects with FH starting from childhood can grant a precocious nutritional and pharmacological intervention, thus notably reducing the risk of cardiovascular events in young adults [8,9]. For decades, cholesterol screening in childhood has been implemented in many countries, according to each country’s health system and policy, so as to try to improve FH diagnosis [10,11,12,13,14,15].

Many countries have set up FH disease registries with the aim of improving the ability to diagnose and treat the disease, but also to increase knowledge and awareness of this genetic cause of early atherosclerosis among healthcare professionals, patients, and institutions [16]. The LIPIGEN (LIpid transPort disorders Italian GEnetic Network) network has been active in Italy for about 10 years, involving specialised centres for the diagnosis and treatment of genetic dyslipidaemias, of which FH is the most represented due to its higher prevalence. Since 2018, the paediatric subgroup (LIPIGEN Paediatric Group), has been established specifically targeting subjects under the age of 18 [17].

The aim of this exploratory survey was to outline what is known about FH as a genetic disease and as a disease to be treated from childhood. We have evaluated the main access characteristics to the lipid clinic, the healthcare professionals referring patients, and the clinical and biochemical criteria are considered when suspecting FH in children and adolescents in the clinical practice.

## 2. Materials and Methods

**Figure 1:**
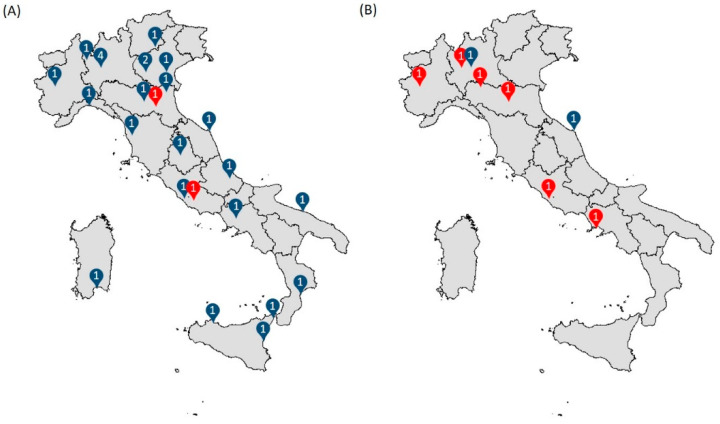
Distribution of the 27 adult lipid clinics that also manage paediatric patients (**A**) and 8 exclusively paediatric clinics (**B**) included in the LIPIGEN Paediatric Group. Centres with at least 40 new paediatric patients per year are in red.

This study employed a cross-sectional survey design to investigate the diagnostic pathways and referral modalities for patients with FH across the 35 Italian lipid clinics included in the LIPIGEN Paediatric Group (Figure 1) [17], which include paediatric clinics (N = 8) or adult lipid clinics that also manage individuals younger than 18 years of age with a suspected FH diagnosis (N = 27).

A structured questionnaire was developed to gather data on the diagnostic procedures and referral patterns for FH patients. The questionnaire consisted of 16 multiple-choice and open-ended questions to explore various aspects related to FH diagnosis and patient pathways (Supplementary Materials). The questions investigated how a paediatric patient with suspected FH is usually referred to the specialist centre and they also assessed the proactive attitude in identifying subjects, such as the type of screening applied to identify subjects with FH among paediatric patients, the parameters measured for the patient’s classification, and the criteria for recommending genetic analysis. The principal investigators were required to choose the answers that better fit with their regular clinical practice.

The survey was administered electronically through a secure online platform. In November 2023, the principal investigator in each centre was invited via e-mail to complete the questionnaire, with an indication to answer the questions by reporting the usual clinical practice of all the medical staff at that centre. Reminders were sent to non-respondents to improve the response rate.

Survey responses were imported into IBM SPSS Statistics for Windows, version 28 (IBM Corp., Armonk, NY, USA). Quantitative data obtained from the survey were analysed using descriptive statistics to examine frequencies and percentages of responses. Qualitative data from open-ended questions were subjected to thematic analysis to identify common patterns and themes.

## 3. Results

The questionnaire was completed by all the invited centres. The centres were spread throughout the country, with 54% in the north, 23% in the centre, and 23% in the south of Italy and the islands.

When asked whether a screening strategy for FH exists in their region, only one centre reported the presence of a regional screening plan, while 8 centres (23%) indicated the presence of a programme but at a local level.

Patient attendance varied widely, with one in three centres reporting fewer than 10 under-18 subjects as first visit per year and 23% reporting more than 40 under-18 subjects as first visit per year; this proportion was higher (75%) among paediatric clinics.

**Figure 2:**
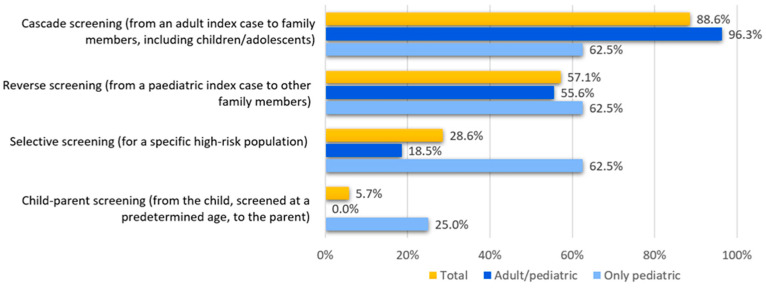
Frequency of responses to the question “At your centre, which screening method for familial hypercholesterolaemia is implemented in paediatric age?”.

The screening method for FH in paediatric subjects that was mainly implemented at the responding centres (Figure 2) was cascade screening (88.6%), followed by reverse screening (57.1%). Selective screening, targeting specific groups of patients at risk, was reported as the screening method in 28.6% of the total respondents. When only paediatric clinics are considered, the percentage rises to 62.5%. Of note, a universal screening strategy was not reported by any of the centres.

Regarding the frequency of use, cascade screening and reverse screening were routinely applied by 62.9% and 71.4% of the centres, respectively, without relevant differences between exclusively paediatric clinics and adult lipid clinics.

Regardless of clinical practice in individual centres, 45.7% of respondents indicated a combination of different approaches as the most appropriate method of FH screening, while 20.0% of them reported universal screening as the preferred method.

**Figure 3:**
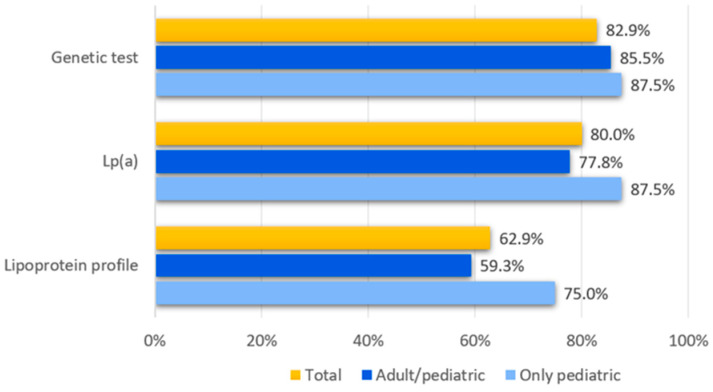
Frequency of responses to the question “At your centre, what biochemical/genetic parameters are required for the detection of familial hypercholesterolaemia?”.

In the characterisation of the FH patients (Figure 3), beyond the lipid profile, genetic testing is used by 82.9% of the centres and lipoprotein(a) [Lp(a)] measurement is used by 80.0%, with a higher percentage (87.5%) for paediatric clinics. The lipoprotein profile is used by 62.9% of the centres, again with a higher percentage (75.0%) for paediatric clinics.

In the decision to refer patients for genetic testing, all centres report LDL-C levels as a guiding criterion. Family history of a cardiovascular event or hypercholesterolaemia is considered in 80.0% and 74.3% of the centres, respectively, while non-response to dietary intervention is a guiding criterion in 25.7% of the centres.

Overall, 80% of the centres reported receiving patients from the paediatrician. Less frequently, they receive patients based on the recommendation of a general practitioner (68.6%) and seldom from a cardiologist (28.6%). One adult centre reported managing paediatric patients as children of adult patients already treated at the centre.

Approximately 30% of centres reported that most patients are referred after being diagnosed with hypercholesterolaemia by their paediatrician. In contrast, a smaller percentage of patients—typically less than 25%—is referred by general practitioners, following cascade screening prompted by the diagnosis of FH in an adult index case (a first-degree relative of the child) or directly by a family member who has been diagnosed with the condition.

## 4. Discussion

This is the second exploratory survey involving the lipid clinics of the LIPIGEN Paediatric Group [17,18]. We evaluated FH screening in the clinical practice and our data confirm that, in Italy, there are sporadic and local screening programmes addressing small populations. When specifically asking which screening method was preferred and used, most of the centres confirmed that cascade screening is the most useful tool to make an FH diagnosis, in line with actual scientific evidence [10]. As a matter of fact, in those countries where FH cascade screening has already been implemented on a national basis, the FH diagnosis rate has considerably increased [13,19]. Reverse screening is often used as well (57.1% of participants), both as a general type of FH screening and as a useful tool in clinical practice. In our survey, centres reported applying cascade/reverse screening in the majority of patients, with no differences between LIPIGEN centres dealing with only paediatric or both paediatric and adult subjects. This evidence confirms the validity of the LIPIGEN network and further highlights the importance of having centres dealing with both paediatric and adult patients at the same site. Almost 30% of centres state that selective screening was implemented predominantly in paediatric centres compared to adult ones (62.5% vs. 18.5%). This response is in contrast with recent scientific evidence [20], and it could be explained by the presence of “historical” centres among the LIPIGEN network that had used this screening strategy decades ago, according to specific past recommendations [19].

When asked about what the most appropriate FH screening strategy is, 45.7% of centres indicated the combination of multiple screening methods, whereas only 20% declared that universal screening is the best option. This response reflects the healthcare landscape in Italy, where implementing systematic screening would demand an economic and organisational investment that is not currently feasible. Indeed, the Prague Declaration states that every country should implement the best FH screening strategy according to each country-specific health system situation and funding [10].

A notable tendency toward conducting detailed analyses of biochemical parameters in defining the suspicion of FH was also observed, along with a frequent reliance on genetic confirmation. All centres require a full plasma lipid profile, and 62.9% of centres also require a full lipoprotein profile (with a higher percentage among paediatric centres). Lipoprotein(a) plasma levels were evaluated in four out of five centres and in more than 87% of paediatric centres (87.5%). These results are far better compared to those described in the literature [16] and are probably due to the constant updates to scientific work carried out among LIPIGEN network centres [21].

The percentage of centres referring their patients to undergo FH genetic testing is 82.9%, slightly higher than what was reported in other European countries [22]: this finding is likely to be attributable to the adherence to the LIPIGEN network which supports this practice [17]. When we looked more closely at the criteria for genetic testing of paediatric patients, we found that LDL-C plasma levels, as well as family history for hypercholesterolemia and premature coronary vascular disease, are considered by 74.3% of centres dealing with adult patients and 80% of those dealing with paediatric ones, and this is in line with the recommendations by most recent consensus documents on paediatric FH [19,23,24].

In the last section of the survey, we tried to identify which health professional figures were involved in referring patients to the specialist centre. As expected, general paediatricians were found to be the main doctors who referred paediatric patients to paediatric lipid clinics [19,25,26]. Indeed, in the absence of a systematic screening programme at the national level, the identification of children with suspected FH mostly occurs incidentally by paediatricians, sometimes following lipid tests prescribed for other reasons. This approach also explains the significant involvement of general practitioners, who, in the Italian healthcare system, can care for individuals as young as six years old. In a small percentage, the general practitioner refers a paediatric patient after diagnosing FH in an adult family member under their care. These results suggest a lack of awareness that FH is a frequent dominant genetic condition [27,28,29], but this could also be due to a lack of knowledge about the existence of specific centres for the diagnosis and treatment of the disease. Not only in Europe but on all continents, the lack of awareness of FH is still one of the major barriers to diagnosis [16], confirming what was already highlighted by the literature [13,14,15].

Approximately 30% of the centres identified cardiologists as the healthcare professionals who referred the patient to the specialised centre. Although this percentage is lower compared to those related to other healthcare professionals, it should be regarded as a warning sign, as it often implies that the cardiovascular risk in these young patients has already manifested in a clinically evident event.

While our study demonstrates that diagnostic performance at the centres is commendable, it also reveals notable inconsistencies in the adoption of screening strategies and in the collaboration between specialist centres, general practitioners, paediatricians, and other specialists. We believe that these discrepancies underscore the urgent need for a common set of guidelines or consensus documents to standardise practices across the network and strengthen the link with primary care providers. Furthermore, beyond the routine surveys conducted among the specialist centres, the LIPIGEN network will implement dedicated surveys for both physicians and patients to enhance awareness of the critical importance of early and accurate disease diagnosis. Promoting awareness and understanding of the condition among all healthcare professionals and within families is crucial to ensuring early diagnosis and timely initiation of therapy, thereby preventing the development of cardiovascular events.

### Strengths and Limitations

The design of a simple and fast-filling questionnaire was certainly an advantage and resulted in optimal adherence (100% of centres participated in this survey). Another strength is the very presence of the network, which has allowed us to interrogate different clinical realities and to obtain a sampling of centres distributed throughout the country, also considering that, in Italy, FH is mainly managed by specialists. However, we have to recognise as a limitation that the answers were collected from medical doctors or researchers at lipid centres; thus, their point of view on this topic could be different from that of general practitioners and primary care paediatricians and from that of patients themselves. As a matter of fact, a survey composed of a questionnaire filled in by both general practitioners and patients would have been very difficult to achieve and it would probably have been less effective to highlight adequate points of interest on this topic in a short time.

## 5. Conclusions

The results of our survey highlighted that FH screening strategies are adequately known and implemented in the clinical practice by most of the involved lipid centres. Despite the lack of a shared systematic screening programme, we have documented robust and refined diagnostic management of paediatric patients with FH, both considering the high rate of Lp(a) plasma level determination and the high percentage of paediatric patients who undergo genetic FH testing. The results of our survey advocate for a great need to improve knowledge of FH and therefore the ability to diagnose and manage this condition starting from childhood; the LIPIGEN Paediatric Group will be actively involved in these issues as well as in the scientific research on this topic.

## Data Availability

The datasets generated and/or analysed during the current study are available from the corresponding author upon reasonable request.

## Supplementary Materials

A full template of the survey can be downloaded at https://www.mdpi.com/article/10.3390/children12030288/s1.

## Abbreviations

FH	familial hypercholesterolemia
LDL-C	low-density-lipoprotein cholesterol
LIPIGEN	LIpid transPort disorders Italian GEnetic Network
Lp(a)	lipoprotein(a)
