# Supplementary material for: Diagnosis and Screening Strategies for Detection of Familial Hypercholesterolaemia in Children and Adolescents in Italy: A Survey from the LIPIGEN Paediatric Group
Source: Children (Basel). 2025 Feb 26;12(3):288. doi: 10.3390/children12030288 (PMC11941534; doi:10.3390/children12030288)
Supplement: Supplementary file 1 [file children-12-00288-s001.zip › children-3468684-supplementary.pdf]

children

Article

Diagnosis and screening strategies for detection of Familial Hypercholesterolaemia in children and adolescents in Italy: a survey from the LIPIGEN Paediatric Group

Supplementary Material

Survey

1. Indicate your affiliated LIPIGEN center:
2. In which region is your center located?
3. Is there a screening program for familial hypercholesterolemia in your region?
   - Yes, regional
   - Yes, local
   - No
4. The center where you operate is a clinic/specialized center for:
   - Both adults and pediatric patients
   - Exclusively pediatric patients
5. How many pediatric patients (under 18 years old) undergo a first visit at your center each year?
   - < 10
   - 10-20
   - 20-40
   - > 40
6. How many pediatric patients (under 18 years old) are currently in follow-up at your center?
   - < 50
   - 50-100
   - 100-200
   - > 200
7. What screening method for familial hypercholesterolemia is implemented for pediatric patients at your center?

   (Multiple answers possible)
   - Universal screening
   - Selective screening (for a specific high-risk population)
   - Cascade screening (from an adult index case to family members, including children/adolescents)
   - Reverse screening (from a pediatric index case to other family members)
   - Child-parent screening (from a child screened at a predetermined age to the parent)
   - Other (please, specify...)
8. In your opinion, what is the most appropriate screening method for familial hypercholesterolemia?
   - Universal screening
   - Selective screening
   - Cascade screening

- o Reverse screening
- o Child-parent screening
- o A combination of different methods
- o Other (please, specify...)

9. In the diagnostic process for pediatric patients with hypercholesterolemia, do you routinely apply cascade screening?
   - o Always
   - o 50%
   - o < 50%
   - o Never
10. In the diagnostic process for pediatric patients with hypercholesterolemia, do you routinely apply reverse screening?
    - o Always
    - o 50%
    - o < 50%
    - o Never
11. At your center, which biochemical/genetic parameters are requested for the assessment of familial hypercholesterolemia?
    (Multiple answers possible)
    - o Lipid profile: Total Cholesterol (TC), HDL-C, LDL-C, Triglycerides (TG)
    - o Lipoprotein profile: ApoA1, ApoB
    - o Lp(a)
    - o Genetic analysis
12. At your center, is Lp(a) evaluation requested for the assessment of pediatric patients with familial hypercholesterolemia?
    - o Always
    - o 50%
    - o < 50%
    - o Never
13. At your center, in cases of clinical suspicion, is genetic analysis requested for the diagnosis of familial hypercholesterolemia in pediatric patients?
    - o Always
    - o 50%
    - o < 50%
    - o Never
14. What are the main criteria you use to request genetic analysis for pediatric patients?
    (Multiple answers possible)
    - o LDL-C levels
    - o Family history of cardiovascular disease (CVD)
    - o Family member with hypercholesterolemia
    - o Poor response to dietary intervention

15. Which physicians most commonly refer pediatric patients to your center?
    (Multiple answers possible)
    - Primary care pediatricians
    - General practitioners
    - Cardiologists
    - Other:
16. What percentage of pediatric patients (under 18 years old) undergoing a first visit at your center are referred by a primary care pediatrician/general practitioner after detecting hypercholesterolemia?
    - < 10%
    - 10-25%
    - 25-50%
    - 50-75%
    - > 75%
17. What percentage of pediatric patients (under 18 years old) undergoing a first visit at your center are referred by a general practitioner treating a parent or first-degree relative with hypercholesterolemia (cascade screening)?
    - < 10%
    - 10-25%
    - 25-50%
    - 50-75%
    - > 75%
18. What percentage of pediatric patients (under 18 years old) undergoing a first visit at your center are referred on the initiative of a family member with hypercholesterolemia?
    - < 10%
    - 10-25%
    - 25-50%
    - 50-75%
    - > 75%
19. You may leave a comment:

Disclaimer/Publisher's Note: The statements, opinions and data contained in all publications are solely those of the individual author(s) and contributor(s) and not of MDPI and/or the editor(s). MDPI and/or the editor(s) disclaim responsibility for any injury to people or property resulting from any ideas, methods, instructions or products referred to in the content.
